# The Regulatory Role of High-Mobility Group Protein 1 in Sepsis-Related Immunity

**DOI:** 10.3389/fimmu.2020.601815

**Published:** 2021-01-22

**Authors:** Li Li, Yuan-Qiang Lu

**Affiliations:** ^1^ Department of Emergency Medicine, School of Medicine, The First Affiliated Hospital, Zhejiang University, Hangzhou, China; ^2^ Department of Geriatrics, School of Medicine, The First Affiliated Hospital, Zhejiang University, Hangzhou, China; ^3^ Zhejiang Provincial Key Laboratory for Diagnosis and Treatment of Aging and Physic-Chemical Injury Diseases, Hangzhou, China

**Keywords:** sepsis, HMGB1, inflammation, pyroptosis, immunosuppression

## Abstract

High-mobility group box 1 (HMGB1), a prototypical damage-associated molecular pattern (DAMP) molecule, participates in multiple processes of various inflammatory diseases through binding to its corresponding receptors. In the early phase, sepsis is mainly characterized as a multi-bacterial-induced complex, excessive inflammatory response accompanied by the release of pro-inflammatory mediators, which subsequently develops into immune paralysis. A growing number of *in vivo* and *in vitro* investigations reveal that HMGB1 plays a pivotal role in the processes of inflammatory response and immunosuppression of sepsis. Therefore, HMGB1 exerts an indispensable role in the immune disorder and life-threatening inflammatory syndrome of sepsis. HMGB1 mainly mediate the release of inflammatory factors *via* acting on immune cells, pyroptosis pathways and phosphorylating nuclear factor-κB. Moreover HMGB1 is also associated with the process of sepsis-related immunosuppression. Neutrophil dysfunction mediated by HMGB1 is also an aspect of the immunosuppressive mechanism of sepsis. Myeloid-derived suppressor cells (MDSCs), which are also one of the important cells that play an immunosuppressive effect in sepsis, may connect with HMGB1. Thence, further understanding of HMGB1-associated pathogenesis of sepsis may assist in development of promising treatment strategies. This review mainly discusses current perspectives on the roles of HMGB1 in sepsis-related inflammation and immunosuppressive process and its related internal regulatory mechanisms.

## Introduction

Sepsis is the body’s maladjusted response to an infection. It is manifest as an excessive systemic, inflammatory immune response in its early stage and as continuous immunosuppression in its later phase. These processes may result in cell dysfunction and ultimately in organ failure (1). Effective sepsis-specific therapies are still lacking. Most current therapeutics focus mainly on supporting dysfunctional organs rather than on cures. Existing therapies include administration of antibiotics to fight against various infections; restoration of fluid balance and use of a vasopressor to maintain perfusion of vital organs; and utilization of mechanical support of failed organs (such as mechanical ventilation in failed lungs, dialysis-like techniques for renal failure, etc.).

Progress has been made on not only in development of technologies but also in comprehensive investigation of the pathogenesis of sepsis and of the diverse interactions between pathogens and hosts. However, sepsis-induced mortality is still unacceptably high typically about 30% and even as much as 40–50% when shock develops (2). In additions, survivors of severe sepsis also show an increased risk of post-sepsis syndrome, such as cognitive impairment and functional disability, which in turn contributes to an elevated risk of various mental and psychological diseases (3).

HMGB1 has been implicated in the pathogenesis of multiple diseases, including cancer, traumatic shock, and autoimmune diseases (4, 5). It has been well proven that systemic HMGB1 levels are markedly increased in murine sepsis models and in patients with sepsis (6). After treatment with lipopolysaccharide (LPS), serum HMGB1 in mice increases from 8 h and peaks in 16 to 32 h (7). The delayed secretion of HMGB1 broadens the time window of treatment opportunities for patients with sepsis using HMGB1 antagonists. Besides, study has shown that reducing the release of HMGB1 can effectively improve the survival of mice suffering from sepsis (8).

In this review, we review some new insights into the roles of HMGB1 associated with inflammation, immunosuppression in sepsis. Understanding the contributions of HMGB1 to the pathogenesis of sepsis may facilitate development of potentially effective therapeutic strategies to shorten the course of the disease as much as possible, and to improve the prognosis and survival rate of sepsis patients.

## Insights on HMGB1

HMGB1, a multi-functional and highly conserved nucleoprotein with 215 amino acid residues, includes two tandems, positively charged DNA-binding regions, A box and B box, together with a negatively charged tail consisting of a string of amino acid residues. The A box has no innate pro-inflammatory activity, but can specifically antagonize the cytokine and pro-inflammatory activities of HMGB1 (9). The B box comprises most of the pro-inflammatory properties of HMGB1. Amino acid residues 89-108 of HMGB1, located in the top 20 of B box, are the main region for stimulating cytokine release (10). Two nuclear localization sites (NLS) in the A and B boxes, respectively, regulate the capacity of HMGB1 to be transported into the cytoplasm. HMGB1 consists of three isoforms: disulfide HMGB1, fully reduced HMGB1 and sulfonyl HMGB1, among these disulfide HMGB1 is the only isoform with the pro-inflammatory activity (11).

As one of the earliest confirmed members of the damage-associated molecular pattern family and a prototypical alarm molecule, HMGB1 initiates and maintains the immune response during inflammation (7). The immune function of extracellular HMGB1 is completely dependent on the redox status of its three conserved cysteine residues, at locations 23 and 45 in box A and at location 106 in box B, that regulate receptor binding ability (12). HMGB1 is translocating outside the cell mainly through two pathways: passive release and active secretion. Passive HMGB1 release mainly occurs in different forms of cell death, for instance, pyroptosis, apoptosis, and necrosis (13). HMGB1 released by different forms of dead cells has different redox states. When emancipated after pyroptosis, it is usually in the disulfide form, after apoptosis in the fully oxidized form and after necrosis in fully reduced or disulfide forms (7). Disulfide HMGB1 and fully reduced HMGB1 can be converted to each other according to the redox state of the internal and external environment of the cell, while fully oxidized HMGB1 is an irreversible form (14).

HMGB1 can be actively released by a variety of cells, including endothelial cells, hepatocytes, macrophages, and monocytes (6). Active HMGB1 release is a complex process. First, transport from the nucleus into the cytoplasm depends on posttranslational modifications of NLS such as acetylation, methylation, and phosphorylation, which may in turn be regulated by the JAK/STAT1 signaling pathway and calcium/calmodulin-dependent protein kinase (CaMK) IV (6). Secondly, cytoplasmic HMGB1 is released to the extracellular environment by induction of programmed pro-inflammatory cell pyroptosis or by exocytosis of secretory lysosome the same pathway as IL-1β secretion (15). HMGB1 is commonly detected in nucleated mammalian cells, and its activity is closely related to its cellular location. During homeostasis, nuclear HMGB1 performs an essential role in maintaining the stability of cell functions through its involvement in multiple processes such as DNA replication, transcription, stabilizing nucleosome formation, and regulating gene expression (16). In response to various injuries and inflammatory stimuli, HMGB1 is transported from the nucleus to the cytoplasm and participates in important pathways, for instance, in promotion of inflammasome activation, initiation of autophagy, and regulation of apoptosis (17–19). Once released to the extracellular environment, HMGB1 attains the vital ability to induce chemokine/cytokine production, stimulate neutrophil extracellular trap formation, involvement in neuroimmune or metabolic activities, and other activities (6). Translocation of HMGB1 from the nucleus to the cytoplasm and ultimate extracellular secretion are also key processes for HMGB1-mediated inflammation. Advanced glycation end products (RAGE) and toll-like receptor 4 (TLR4) are important members of the innate immune system (20). HMGB1 exerts its biological function mainly through binding to any of these two receptors (21).

## The Role of HMGB1 in Inflammation

Pro-inflammatory cytokines, released from various immune cells, are considered to be essential mediators in the lethal effects of endotoxins. Excessive release of cytokines is closely associated with tissue damage, multiple-organ dysfunction or failure, and even death (22). As an effective pro-inflammatory cytokine, HMGB1 plays a role in various inflammatory diseases, especially in sepsis. In both experimental sepsis models and in sepsis patients (7, 11, 12), the levels of HMGB1 in circulation are remarkably increased, and the concentration of circulating HMGB1 is positively correlated with the severity of inflammation and disease. HMGB1 can also induce inflammatory anemia and cognitive impairment, which is considered to be related to neuroinflammation, among sepsis survivors (15, 23). Compared with other early -phase pro-inflammatory contents, for instance tumor necrosis factor (TNF) and IL-1β, HMGB1 began to appear 8 h and significantly increased after initiation of sepsis (9). Thus, HMGB1 offers a relatively wider time window for clinical treatment of sepsis patients with progressive inflammation.

The early stage of sepsis is accompanied by excessive inflammation, which is the main initial actor of organ dysfunction. Because continued inflammation can have serious consequences, including organ failure, it seems a reasonable therapeutic goal to the auto-inflammatory response to avoid excessive activation of the immune response and a cascade of effects that induce cell and tissue damage. Earlier studies have demonstrated that blocking strong expression of HMGB1 in inflammatory diseases, such as rheumatoid arthritis, myositis, and systemic lupus erythematosus can attenuate inflammation (24). Recent studies have found that targeting HMGB1 can reduce inflammatory response which in turn reduces sepsis associated organ damage (25–28). Therefore, antagonizing HMGB1 in serum or intervening in the pathway of HMGB1 mediated pro-inflammatory pathways may facilitate development of potential therapies in sepsis.

## HMGB1 and Immune Cells

**Figure 1:**
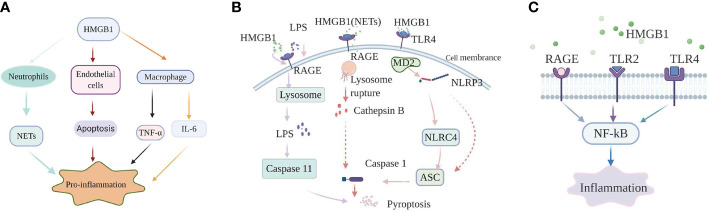
High-mobility group box-1 (HMGB1)-associated regulatory pathways of inflammation in sepsis. **(A)** HMGB1 and immune cells. HMGB1 promote NETs formation of neutrophil, ECs apoptosis to mediate inflammation; HMGB1 can also directly promote macrophages to release inflammation factors, such as: TNF-α, IL-6. **(B)** The role of HMGB1 in pyroptosis. Extracellular HMGB1 induces both canonical and noncanonical pyroptosis pathways by binding to their corresponding receptors. **(C)** HMGB1-RAGE/TLRs-NF-κB signaling pathways. HMGB1 binds to receptors, containing receptor for advanced glycation end products (RAGE), Toll-like receptor 2 (TLR2), and Toll-like receptor 4 (TLR4) on the cell membrane, to phosphorylate nuclear factor-κB, which regulates the generation of inflammatory mediators.

HMGB1 promotes sepsis-induced organ dysfunction through suppressing neutrophil ability to clear bacteria, and so enhancing persistent inflammation. In both mice subjected to sepsis and patients surviving septic shock, HMGB1 decreased the capacity of neutrophil to kill bacteria through mediating neutrophil nicotinamide adenine dinucleotide phosphate (NADPH) oxidase dysfunction (21). Interestingly, in an abdominal sepsis mice model, Zhou et al. manifested that platelet-derived HMGB1 facilitated neutrophil activation and reactive oxygen species (ROS) generation, which were critical for the ability of neutrophil to promote bacterial clearance (29). These contradictory results indicate that the functions of HMGB1 in sepsis are complex and different. Perhaps the final balance of these effects determines whether the role of HMGB1 in sepsis is beneficial or harmful. Additionally, investigation showed that platelet-derived HMGB1 stimulated neutrophil extracellular traps (NETs) formation in septic shock patients and sepsis mouse model induced by cecal ligation and puncture (CLP) (30). Research on an abdominal septic animal model also showed that levels of NETs were significantly increased. These increased NETs could recruit neutrophils to the lung and promote the formation of pro-inflammatory compounds in pulmonary (31). Apart from this effect, NETs could also stimulate macrophages to secrete massive inflammatory mediators, tumor necrosis factor-α (TNF-α), HMGB1, which boost systemic inflammation (31). In an animal model of pulmonary fibrosis, interaction of HMGB1 with phosphatidylserine on the surfaces of apoptotic neutrophils inhibited the engulf of apoptotic neutrophils by macrophages (32). Therefore, apoptotic neutrophils remained to generate pro-inflammatory contents of reactive oxygen species (ROS), which aggravated the pulmonary pro-inflammatory environment and enhanced the lung tissue injury. Whether HMGB1 plays the same role in sepsis is question that requires additional investigation. As a member of innate immune cells, macrophages act a vital role in initiating body’s immune response. Studies on both patients with sepsis and sepsis animal model indicated that HMGB1 could facilitate the release of inflammatory cytokines from macrophages, such as: TNF-α, interleukin-6 (IL-6) (33). However, the specific internal regulation mechanism is still unclear. HMGB1 can not only promote the release of inflammatory factors from macrophages, but also facilitate the conversion of macrophages to an inflammatory phenotype. In experimental mouse model of autoimmune myocarditis, HMGB1 promoted macrophage transforming into M1-like Phenotype owning pro-inflammatory activity *in vitro* (34). Loss of endothelial cells (ECs) integrity can cause inflammatory substances to leak into surrounding tissues, and so facilitate tissue damage and inflammation (35). HMGB1 in circulation from patients subjected to sepsis promoted ECs apoptosis, which in turn increased endothelial (36). The Internal mechanism may be related to the expression levels of some proteins mediated by HMGB1 (**Figure 1A**).

## HMGB1 and Pyroptosis

In sepsis, pyroptosis is a special form of programmed cell death, which is accompanied by release of multiple inflammatory compounds, including IL-18 and IL-1β. Normal pyroptosis defends the host from bacterial infection and reduces organ damage. However, excessive pyroptosis can lead to multiple organ dysfunction or failure and septic shock, or to increased chances of secondary infection (37, 38). Pyroptosis contains two chief pathways: one canonical, caspase 1-dependent pathway, and a noncanonical pathway associated with caspase4/5 and caspase11 (39). Caspase4/5 mainly regulates human-related pyroptosis, while caspase11 mainly participates in animal-related pyroptosis.

The nucleotide binding and oligomerization domain-like receptor family pyrin domain-containing protein 3 (NLRP3) inflammasome, a molecule is highly sensitive to both DAMPs and pathogen-associated molecular patterns (PAMPs). When oxidized to form cysteine 23-cysteine 45 bonds, HMGB1 can signal through TLR4-MD2 and act as a priming factor for NLRP3 inflammasome activation (40). When activated by HMGB1, NLRP3 can activate caspase-1 through recruiting nucleotide-binding domain and leucine-rich repeat (NLR) family, caspase recruitment domain (CARD) containing 4 (NLRC4), apoptosis associated speck like protein (ASC), and finally initiating a canonical pathway of pyroptosis and prompting the production of inflammatory factors in sepsis (41). Other research on sepsis model also indicated that hepatocyte-derived HMGB1 transported LPS into the cytosol of macrophages and endothelial cells, *via* RAGE-mediated internalization (23). Subsequently, LPS activated a noncanonical pyroptosis pathway induced by caspase-11, which in turn increased inflammation and the lethality of sepsis (23). Furthermore, neutralizing extracellular HMGB1 or inhibition of HMGB1-LPS binding could prevent caspase-11-dependent pyroptosis and death in endotoxemia (23). Caspase-11-mediated pyroptosis of renal tubular epithelial cells is also a pivotal point during septic acute kidney injury (42). However, caspase-11 knockout can attenuate pathological kidney damage and improve survival, in both *in vitro* and *in vivo* experiments in mice (42). In addition, investigation also revealed that HMGB1 alone initiated ASC-dependent and caspase-11-independent pyroptosis (43). Yang et al. (44) indicated that in hemorrhagic shock, HMGB1 stimulates lung endothelial cell (EC) endocytosis through the RAGE receptor, which in turn facilitates the activation and release of cathepsin B from ruptured lysosomes. This process is then followed by the formation of pyroptosomes and activation of caspase-1, leading to EC pyroptosis and increased lung damage (44). The lung is the most readily targeted organ during the process of sepsis. Thus, it would be worthwhile investigating whether the pathway plays the same role in sepsis and whether manipulation of that pathway could provide an effective intervention. Similarly, in a septic mouse model, NETs-derived HMGB1 induced macrophage pyroptosis through the same signaling pathway demonstrated by Yang et al. (45). Recent investigation performed by Wang et al. (46) manifested that HMGB1 acted as an vital intermediary molecule in the process of TNF-α induce pyroptosis in M1 macrophages in the animal model of acute liver failure induced by LPS (**Figure 1B**).

## HMGB-RAGE/TLRs-NF-κB Signaling Pathways

HMGB1 exerts its pro-inflammatory effects mainly by binding to multiple membrane receptor proteins, including TLR2, RAGE, and TLR4 (16). The important role of the HMGB1-RAGE/TLRs axis is to phosphorylate nuclear factor-κB (NF-κB), facilitating secretion of various proinflammatory cytokines (47).

In sepsis-induced animal model of lung damage, study show that the protective role of Xuebijing (XBJ) may be *via* inhibiting HMGB1/RAGE axis to decrease pro-inflammatory cytokines releases (48). Additionally, literature documented the combination of HMGB1 and RAGE can trigger a series of signal transduction pathways, which can directly activate NF-κB or indirectly through the mitogen-activated protein kinase (MAPKs) pathway or other ways, thereby promoting the release of inflammatory factors (49). However, the specific details of the activation of the NF-κB pathway by the HMGB1-RAGE axis are not yet clear in sepsis. Moreover, multiple studies on animal models of sepsis found that suppression HMGB1/TLR4/NF-κB signaling pathway could attenuate inflammation response and ultimately reduce the damage of vital organs as well (50, 51). It is worth noting that in a sepsis rat model, serum-derived HMGB1 accumulates in the renal tissue and urine and turn renal tubular epithelial cells (TECs) into inflammatory promoter mediators, which facilitate the release of pro-inflammatory cytokines through binding to TLR4. Moreover, The process accompanied by the activation of MAPK and NF-κB as well (52). Apart from this, study also show that TLR4 and TLR2 receptors convey signals through a MyD88-dependent/independent pathway, which can activate NF-κB (53, 54).

The studies listed above all show that HMGB1 can mediate inflammation response in sepsis by binding to RAGE or TLRs. However, literature also report if TLR4 is functionally inactivated or absent, macrophages containing both RAGE and TLR4 receptors, cannot generate cytokines when initiated by any isoforms of HMGB1 (55). Besides, in LPS-induced mouse model of acute lung injury (ALI), HMGB1 can simultaneously activate the TLR4, TLR2, and RAGE/NF-κB signaling pathways to promote the activation of absent in melanoma 2 (AIM2) inflammasome in macrophage and the polarization of M1 macrophages, followed by production of interleukin−1β (IL-1β) and IL-6 (56). Therefore, the specific mechanism of HMGB1/TLRs/RAGE/NF-κB pathways that mediate inflammation in sepsis need further exploration (**Figure 1C**).

Therefore, HMGB1 participates in the release of pro-inflammatory factors in sepsis in a variety of ways. Whether these pro-inflammatory pathways affect each other or which is the dominant pathway and the specific regulation process of these pathways need further study.

## The Role of HMGB1 in Immunosuppression

It is well known that following an early cytokine storm, late phase sepsis is always accompanied by long-term chronic inflammation and sustained immunosuppression (57, 58). Patients with severe sepsis have a long course of disease and usually exhibit persistent inflammation-immunosuppression and catabolic syndromes, leading to many adverse clinical consequences. The specific mechanisms of the syndrome are currently unknown. However, increasing evidence indicates that alteration of myelopoiesis, expansion of immature myeloid-derived suppressor cells (MDSCs), and reduction of effector T-cell function, are all contributors to the immunosuppressive pathology of sepsis. HMGB1 plays a unique role in the development of post-sepsis immunosuppression. Dynamic monitoring indexes of HMGB1 and cellular immunity are vital for assessing chronic inflammation processes, immune status, and prognoses of patients with severe sepsis (59). However, a consensus on how HMGB1 applies its immunomodulatory function in sepsis remains to be further demonstrated.

## HMGB1 and Neutrophils

High level of HMGB1 acts as a specific role in the development of immunosuppression during late phase of sepsis. Research revealed that HMGB1 could inhibit NADPH oxidase activation, leading to a deficiency in oxidative burst and dysfunction of neutrophil-dependent bacterial killing mechanisms in an experimental sepsis model and in patients surviving septic shock (21). Additionally, these neutrophils with immunosuppressive phenotypes not only participate in the development of late-phase immunosuppression, but also affect newly generated neutrophils.

Early research also demonstrated that HMGB1 binding to RAGE receptor could effectively inhibit NADPH oxidase activation in neutrophils (60). However, the mechanism needs to be further confirmed in sepsis.

## HMGB1 and MDSCs

Decreasing the numbers of immune cells caused by apoptosis of lymphocytes and imbalance of immune function is one of the main mechanisms of immunosuppression in sepsis (61). Myeloid-derived suppressor cells (MDSCs) have recently been correlated with the profound immunosuppression in sepsis and can suppress T-cell proliferation *via* a variety of mechanisms (62). MDSCs not only inhibit effector T cells, but they also mediate the expansion of regulatory T cells (Tregs) a recognized type of immunosuppressive cell under inflammatory conditions (63). One important factor is that release of ROS molecules is also one of the main mechanisms by which MDSCs inhibit T cells (64, 65).

Generation of ROS is essential not only for the immunosuppressive function of MDSCs, but also seems to keep these cells in an undifferentiated state (66). ROS is one of the critical regulators in the control of HMGB1 release (5). However, whether ROS derived from MDSCs promotes the production of HMGB1 needs further verification. In the tumor micro-environment, HMGB1 maintains the survival of MDSCs through facilitating autophagy, which promotes the potency of MDSCs to restrain anti-tumor immunity and accelerate tumor progression (67). Thus, it is meaningful to speculate whether HMGBI promotes the survival of MDSCs and maintains the immunosuppressive function of MDSCs *via* inducing autophagy of MDSCs in sepsis. Li et al. suggested that in tumors, the release of HMGB1 mediated by inflammasomes further induces the expression of CD274/PD-L1, which results in immunosuppression (68). T-cell depletion in sepsis is caused mainly by programmed cell death-1 (PD-1) interaction with its ligand (PD-L1), and the use of antibodies to PD-L1 can improve the survival rate in sepsis (69). Moreover, investigation recently show that MDSCs might act a immunosuppressive effect *via* PD-L1/PD-1 axis in sepsis mouse model (70). Whether any of these molecules are mediated by HMGB1 or whether HMGB1 promotes the survival of MDSCs through other specific pathways or enhances its immunosuppressive capacity in sepsis is still unknown and needs further research. There have been many reports about MDSCS inhibiting T cell proliferation and function. However, few investigations have focused on the role of MDSCs on B-cells. Recent research shows that human M-MDSCs, significantly inhibits function of human B cells and alters the subsets of B-cell *in vitro* (71). Therefore, MDSCs could play an important role in immunosuppression, which might be caused by simultaneously inhibiting the functions of T cells and B cells. Thence, exploring the role between HMGB1 and MDSCs is of great significance in discovering new sepsis-related immunotherapy strategies.

In contrast, Liu et al. have concluded that HMGB1 could reverse immunosuppression in sepsis, when released at moderate levels (72). Only when HMGB1 is over-released can it facilitate immune paralysis. Furthermore, once activated by caspase-1, HMGB1 can antagonize apoptosis-induced tolerance through the RAGE signaling pathway in dendritic cells (61). Thence, Whether HMGB1 exerts immunosuppressive effects may also be related to the concentration and activation mode of HMGB1.

## Conclusion

HMGB1 is a contradictory molecule in sepsis. It not only mediates inflammation response, but also plays an important role in the immunosuppression of sepsis. In this review, we mainly introduce the relevant pathways of HMGB1 in mediating inflammation in sepsis. At present, there are relatively few studies on the immunosuppressive effect of HMGB1 in sepsis. However, the immunosuppressive effect of MDSCs in sepsis and the related mechanism have been preliminary studied. Exploring the possible regulatory mechanism between HMGB1 and MDSCs in sepsis may provide new ideas for the immunotherapy of sepsis. It is regrettable that there are relatively few investigations on when HMGB1 acts a pro-inflammatory or immunosuppressive effect in sepsis. Therefore, in order to provide more accurate targeted therapy for sepsis, more detailed studies on HMGB1 are needed.

## Author Contributions

LL conceived and completed the manuscript. Y-QL supervised and revised the manuscript. All authors contributed to the article and approved the submitted version.

## Funding

This review was supported by the grants from the Opening Foundation of State Key Laboratory for the Diagnosis and Treatment of Infectious Diseases (Nos. 2018KF02, and SKLID2019KF06), the National Natural Science Foundation of China (Nos. 81272075 and 81801572), and the Foundation of Key Discipline Construction of Zhejiang Province for Traditional Chinese Medicine (No. 2017-XK-A36).

## Conflict of Interest

The authors declare that the research was conducted in the absence of any commercial or financial relationships that could be construed as a potential conflict of interest.
